# Embodiment and regenerative implants: a proposal for entanglement

**DOI:** 10.1007/s11019-024-10199-7

**Published:** 2024-03-16

**Authors:** Manon van Daal, Anne-Floor J. de Kanter, Karin R. Jongsma, Annelien L. Bredenoord, Nienke de Graeff

**Affiliations:** 1 Department of Bioethics and Health Humanities, Julius Center for Health Sciences and Primary Care, University Medical Center Utrecht, Utrecht University, Utrecht, The Netherlands; 2 Erasmus School of Philosophy, Erasmus University Rotterdam, Rotterdam, The Netherlands; 3 Department of Medical Ethics and Health Law, Leiden University Medical Center, Leiden University, Leiden, The Netherlands

**Keywords:** Embodiment, Lived experience, Implants, Entanglement, Regenerative medicine, Phenomenology, Incorporation

## Abstract

Regenerative Medicine promises to develop treatments to regrow healthy tissues and cure the physical body. One of the emerging developments within this field is regenerative implants, such as jawbone or heart valve implants, that can be broken down by the body and are gradually replaced with living tissue. Yet challenges for embodiment are to be expected, given that the implants are designed to integrate deeply into the tissue of the living body, so that implant and body become one. In this paper, we explore how regenerative implants may affect the embodied experience of implant recipients. To this end, we take a phenomenological approach. First, we explore what insights the existing phenomenological and empirical literature on embodiment offers regarding the experience of illness and of living with regular (non-regenerative) implants and organ transplants. Second, we apply these insights to better understand how future implant recipients might experience living with regenerative implants. Third, we conclude that concepts and considerations from the existing phenomenological literature do not sufficiently address what it might be like to live with an implantable technology that, over time, becomes one with the living body. We argue that the interwovenness and intimate relationship of people living with regenerative implants should be understood in terms of ‘entanglement’. Entanglement allows us to explore the complexities of human-technology relations, acknowledging the inseparability of humans and implantable technologies. Our theoretical foundations regarding the role of embodiment may be tested empirically once more people will be living with regenerative implants.

## Introduction

For centuries, medicine has aimed to develop treatments to ‘repair’ body parts and improve the lives of those who are ill or injured. Recent advances in bioprinting and stem cell technology could result in treatments that *regenerate* (i.e. regrow) living cells, tissues or organs, which should—ultimately—cure the physical body. These advances are referred to as Regenerative Medicine. A new development emerging in the Regenerative Medicine field are synthetic implants that can be broken down by the body and replaced by living tissue. We refer to these novel implants as *regenerative implants*.^1^ Examples are regenerative jawbone implants for the treatment of (jaw)bone loss and defects, and regenerative heart valve implants for the treatment of heart valve disease.

Within Regenerative Medicine, as in medicine more broadly, the body is seen as an object that can be repaired, restored, and replaced, and there is little attention to the body as the locus of human experience (Carel 2011; Derksen 2008; Slatman 2014b). If Regenerative Medicine lives up to its promises, it might be successful in curing the physical body. Yet the broader aim of curing disease is to improve people’s quality of life, and this requires attention to more aspects of people’s lives than just the health and functioning of the physical body. Attention should also be paid to the everyday life of people and their relationship to their bodies. Moreover, an understanding is needed of how the lives of people are affected by the experience of illness and by (their experience of) the regenerative treatments that they receive. In other words, we need a better understanding of the lived experience and *embodiment* of the people whose physical bodies could be cured with Regenerative Medicine, and with regenerative implants in particular.

As we will explain in more detail below, embodiment refers to the experience of being a body and having a body. A better understanding and acknowledgement of embodiment has the potential to improve healthcare and treatment outcomes (Hudak et al. 2007). For example, for regular knee implants, it is known that the embodied experience of the recipient can play a role in the therapeutic outcomes of the treatment. About one-fifth of the recipients of these implants remain dissatisfied with their new knee. Moore et al. showed that many of the recipients lack an embodied connection to their implant and find it challenging to accept their implant as a normal part of their body (Lape et al. 2019; Moore et al. 2022). They propose that attention to embodiment could facilitate the rehabilitation process. For example, attention to the new relationship between the individual and their new body part could help the recipient to accept their implant.

Until now, the importance of embodiment for Regenerative Medicine has received little attention.^2^ Yet also for this field challenges related to embodiment are to be expected. This is especially true for regenerative implants, as they are designed to integrate deeply into the tissues of the living body, so that implant and body become one. Earlier, we have argued that it is conceivable that the intimate integration of these regenerative implants into the tissues of the body could affect the lived experience of recipients (De Kanter et al. 2023a; Van Daal et al. 2023).

In this paper, we explore in depth how regenerative implants may affect the embodied experience of implant recipients. To this end, we take a phenomenological approach. Phenomenology is the tradition in philosophy that analyses the structure of lived experiences, i.e. how phenomena appear to us in our consciousness, and philosophers in this tradition have explored embodiment and the first-person experience of living with and in a body in depth.

First, we explore what insights the existing phenomenological and empirical literature on embodiment offers regarding the experience of illness in general and of living with regular^3^ implants in particular. We elaborate on the notion of embodiment and draw on related notions of transparency and incorporation, which have been used by phenomenologists to describe and conceptualize the lived experience of illness and regular implants, as well as to describe the relationship between the body, technology, and the world (Carel 2011; Derksen 2008; Ihde 1990, 2002). In our analysis, we draw particularly on the work of Jenny Slatman, who has extensively explored how people deal with living with strange elements as part of their body in the context of limb and organ transplants and prostheses. Second, we apply these and other insights from the existing literature to better understand how people in the future might experience living with *regenerative* implants. Regenerative implants raise the question how people can come to accept a technology as part of their body and learn to experience it as a part of themselves. Third, we argue that concepts and considerations from existing phenomenological literature do not sufficiently address what it might be like to live with an implantable technology that, over time, becomes one with the living body. Regenerative implants add a new dimension of interwovenness which asks for a concept that describes the fusion between regenerative implants and their recipient. Therefore, in the last part of this paper, we argue that the interwovenness and intimate relationship of people living with regenerative implants should be understood in terms of ‘entanglement’. Entanglement allows us to explore the complexities of human-technology relations, acknowledging the inseparability of humans and implantable technologies.

With our analysis, we aim to lay down clear theoretical foundations regarding the role of embodiment in regenerative implants that can be tested empirically once more people will be living with regenerative implants. Our insights can help to make sure that regenerative implants that cure the physical body will also improve the lived and everyday experience of the people who will be living with these implants.

## Embodiment and the experience of living with regular implants

### Embodiment, transparency and incorporation

Human perception and experience are always *embodied*. In other words, our body is a fundamental condition of our experience and the basis for interacting with the world (Dale and Latham 2014; Carel 2011; Merleau-Ponty 2012; Slatman 2014b; Zeiler 2022). This observation finds it roots in the early works of Maurice Merleau-Ponty (Toadvine 2023). He criticized the then prominent phenomenology which disregarded the role of the body in our experience. He argued that there is no human experience that takes place without a body, and pointed to the unity of body and mind directed towards the world (Dale and Latham 2014; Carel 2011; Merleau-Ponty 2012).

We experience our bodies as both object and subject. These two ways of how the body appears in the lived experience were first described by Edmund Husserl as ‘Körper’ and ‘Leib’ (Slatman 2014b; Tbalvandany et al. 2018). Körper means ‘having a body’ (or object body) and refers to the experience of the body as a thing, a fleshy substance in the physical world. Leib means ‘being a body’ (subject body) and refers to the experience of the body as that which is familiar to us, a subject, our means to experiencing the world. For Husserl, embodiment^4^ is inherently two-fold: we both *are* a body, and we *have* a body, and the body is lived as well as material, subject as well as object (Lape et al. 2019; Wehrle 2020). Merleau-Ponty calls this the lived body, the lived unity of mind–body-world system (Bullington 2013; Merleau-Ponty 2012).

During periods of health, we usually do not notice the body. Our body moves to the background of our awareness. In other words, the body is *transparent* (Carel 2011; Dalibert 2015). In contrast, during periods of illness or dysfunction, we become aware of the physical presence of the body, and we experience the body as Körper. In this state, the body becomes either hyperpresent or feels alienated (Lape et al. 2019). For example, physical sensations such as hunger can bring the body to the foreground of our awareness, making it hyperpresent. This awareness of the body and lack of transparency can disrupt the lived unity of mind–body-world system. However, it should be noted that awareness of the body is not necessarily a negative experience. Feminist scholars have pointed out that there are many situations in which we actively experience our body as present, for example during toilet visits, while doing sports, when breastfeeding or when having sex (Derksen & Horstman 2008; Zeiler 2010). These are everyday experiences that can be very enjoyable. Awareness of the body as Körper is therefore not necessarily a negative experience, but it can be, especially in case of illness and injury.

In everyday life, objects or technologies can be used as if they were part of the body itself. In this case, the object (outside or inside the body) becomes part of the *body schema*. The body schema, as introduced by Merleau-Ponty, is the basis for our habitual behaviour, i.e. how we navigate the world in everyday life (Coolen 2014; Merleau-Ponty 2012). It is characterized by an experience of body parts as one dynamic unity (one body) with and within its environment (the world) (Slatman 2014b, p. 66). For example, when we are learning to ride a bike, the bike feels uncomfortable and strange at first. We might act clumsily and may even fall off our bike. After a certain amount of time, the bike starts to feel as if it were an extension of our body. At that moment the bike becomes part of our body schema, and we can cycle without being aware of the bike. This process of integrating something (an object or a habit) into one’s body schema as it is lived in everyday life is called *incorporation* (Merleau-Ponty 2012; Moore et al. 2022). These newly acquired skills and habits are enacted spontaneously, without the need for conscious reflection (Merleau-Ponty 2012).

Don Ihde further explores the concept of incorporation, particularly in how technologies mediate our experiences. For Ihde, incorporating technologies into the body schema is one of the ways in which an (external) tool can mediate the relation between the human and the world (mediated intentionality), like when one wears glasses to see the world more clearly (Ihde 1990; Verbeek 2008). Ihde calls this the ‘embodiment relation’. He emphasizes that in embodiment relations, a person can become unaware of the presence of a technology, as it integrates into their being-in-the-world and becomes transparent (Derksen & Horstman 2008; Ihde 1990, 2002). A technology is characterised by transparency as it withdraws from the forefront or our attention, allowing us to direct our attention to the activity for which the technology is used. In the case of wearing glasses, we can hold a clear view on the world while being unaware of the glasses themselves. When an object is transparent, the technology successfully becomes part of the body, i.e. the body schema. Transparency is lost when the object or technology stops being lived as an inherent part of a person’s relation to the world (Derksen & Horstman 2008). At this point, attention shifts from the ongoing activity involving the person-object dynamic to the tool as a distinct object (Derksen & Horstman 2008).

### Living with injury, illness, and implants

Literature shows that living with an implant can affect the embodied experience of people. Three different phases of the illness narrative are relevant in this context: the initial period of illness or injury, the surgical intervention, and the post-surgery period when the recipient lives with the implant (Lape et al. 2019).

First, most people who need an implant have a long history of illness. People who need a bone implant may, for instance, have a history of rheumatoid arthritis or osteoarthritis, and this history might be marked by unsuccessful treatments. Suffering from such an illness as well as immediate injuries can be a life-changing experience that can disrupt the lived unity of body-mind-world system and evoke feelings of alienation. During illness or injury, we can no longer inhabit and navigate the world, e.g. perform our habits and routines, as we used to do (Carel 2011) and we might perceive the world differently. For instance, illness can make us aware of our body as a medical object (such as on scans and graphs, and through medical information about functionality). In other words, illness or injury can transform our previously taken-for-granted body-world relation (Zeiler 2022). The dysfunctional body-world relationship can be healed through medical treatments, such as an implant, but also through psychological coping mechanisms, which can help people to adapt to illness and injury (Lape et al. 2019).

Second, the surgical intervention required to put an implant in place could be experienced as a threat to bodily integrity and could therefore (temporarily) disrupt the lived unity of body-mind-world system and bring the experience of the body as Körper, as something that can be repaired, to the foreground (Lape et al. 2019). This might be minimized by using less invasive implantations methods. For example, some heart valve implants under development can be implanted through a minor incision and unfold only after they have been put in place (e.g. transcatheter aortic valve replacement). Moreover, it is likely that guidance and support prior to surgery could improve the embodied experience of the surgical intervention (Lape et al. 2019).

Third, living with the implant itself could affect the embodied experience of recipients. Importantly, if the implant is successful in reducing illness symptoms and improving overall wellbeing of a person, this will positively affect their embodied experience. Interventions such as hand transplant surgeries can reduce feelings of alienation and can restore the lived body (Lape et al. 2019; Slatman & Widdershoven 2010). Moreover, some implants make one aware of the body, i.e. cause a loss of transparency, while still affecting the embodied experience positively. For example, spinal cord stimulation, applied through a lead implanted in the spinal cord, is used to eliminate (chronic) pain, but instead causes a tingling feeling called paraesthesia. Both pain and paraesthesia cause the body to be experienced in the foreground of awareness. However, paraesthesia is not necessarily an unpleasant experience. Spinal cord stimulation does not make the body become transparent, but it nevertheless improves the recipient’s embodied experience (Dalibert 2015).

The implant can also evoke negative sensations and emotions in relation to the body part that is malfunctioning. The body can come hyperpresent when the implant causes pain or discomfort or when the implant makes sounds or is visible under the skin (Derksen & Horstman 2008; Jarrett et al. 2009; Lape et al. 2019). Living with an implant can also draw attention to the body when the recipient is forced to change their lifestyle. For example, people with mechanical heart valve implants are required to take blood thinners, which can cause serious bleedings. This means that they can no longer take part in certain sports like boxing, which could draw attention to the body as fragile and falling short (Derksen & Horstman 2008). Additionally, the implant can cause feelings of alienation towards the body, for example if the recipient does not trust the implant or does not consider the implant to be part of themselves (Lape et al. 2019; Moore et al. 2022). In these cases, both the implant and the body are not lived transparently, which may disrupt the body-mind-world system.

In contrast to what some have assumed (De Preester 2010, p. 121), implants do not become incorporated simply by being implanted under the skin and disappearing from view. Rather, the incorporation of strange elements such as implants is an active process, carried out by the person receiving the implant (Dalibert 2015). This raises the question how people can come to accept a technical device as part of their body and learn to experience it as part of themselves. Below, we turn to the work of Slatman whose work has shed light on this question.

### Conditions for tolerating the strange

In her book ‘Our Strange Body’, Slatman explores how people can tolerate the presence of ‘strange’ elements in their bodies (Slatman 2014b). She focusses on the experience of people living with limb and organ transplantations and people living with prostheses.

Slatman argues that people are potentially able to cope well with the addition of external strangeness to the body, such as is the case with prostheses, organ transplants or other types of implants (Slatman 2014b). This is because the body inherently possesses an element of strangeness. Or rather, the body is always familiar and strange at the same time. Slatman’s understanding of the body as familiar (or: ‘own’) and strange at the same time is related to her understanding of the body as Leib and Körper. According to Slatman, the body as a Körper is inherently strange. It is not exclusively or privately ours because it is visible and present to others also. We can only have an experience of this body as *our* body (Leib), because our body has a presence in the physical world where it is visible to others (Körper). Our body as a Leib presupposes our body as Körper. Therefore, our own body is always strange to us to a certain extent (Slatman 2014b). Changes can be made to the physical aspect of our body (our body as Körper) without us necessarily losing the sense that this is our body (our body as Leib). Therefore, we can come to experience the strange elements added to the body as ‘ours’. At the same time, strange elements will also maintain their physical presence, and therefore will remain partly strange, just like the rest of the body (Slatman 2014b, p. 158).

This raises the question: what facilitates the toleration of strange elements in the body? Slatman discusses two conditions that are necessary for tolerating strange elements added to the body (Slatman 2014b, pp. 80, 163). First, the strange body part should be *functionally* adjusted to the body. Second, the strange element must also be *emotionally* or *affectively* tolerated by the person who undergoes the bodily change. Below, we interpret the case studies discussed by Slatman^5^ to explore both conditions in relation to regular implants and limb or organ transplants. We first reflect on both conditions separately, and then shortly discuss the interplay between the two.

#### A. Functional adjustments

The first condition that needs to be met to tolerate strange elements in the body is functionality. This is the condition that healthcare professionals tend to be most focused on. The *functional* condition for tolerating strangeness refers to the need for functional adjustment of the localized sensations.^6^ These include the sense of touch, but also pain, feeling hot and cold, the internal sensation of feeling one’s own body posture (proprioception) and body movement (kinaesthetic sensations) (Slatman 2014b, pp. 71, 161). As Slatman mentions, proprioception literally translates to ‘perception of one’s own’. Having localized sensations in or around a strange element added to the body helps to experience it as part of ourselves (as ‘me’).

Having localized sensations helps for a strange element, such as an implant, to become part of the *body schema* (Slatman 2014b, p. 81). When the implant is functionally adapted to the body, we experience localized sensations that we recognize as our own, and the implant does not require explicit attention. For example, if someone receives a hip implant, over time the sensation of the implant in response to body movements becomes familiar, and with time the person can walk again without having to think about the implant consciously. The process of incorporating the implant into the body schema, and learning how to live with it, is time-consuming (Slatman 2014b). This means that recipients need time and training to become at ease with their new body part.

#### B. Affective tolerance

The second condition that needs to be met to tolerate strange elements in the body is *affective* tolerance*.* Affective tolerance refers to the emotions and feelings that are associated with tolerating the strange. Slatman (2014b) discusses these emotions particularly in relation to the *body image*. Based on Slatmans’s discussion, we distinguish four different elements in the process of affective tolerance. First, Slatman argues that, to tolerate strange elements on an emotional level, one should be able to love and cherish one’s own mirror image. She illustrates this with an example of someone who underwent a successful hand transplant and regained almost full functionality after the transplantation, without visible scarring or colour differences. Nonetheless, this person still felt great distance to the hand and to how it looked on their body. Eventually, they asked the surgeon to amputate the transplanted hand. Apparently, this person could not live with their new mirror image (Slatman 2014b).

Second, our body image is shaped not only by the image of ourselves in the mirror, but also by other people who observe us and to whom we mirror ourselves (Slatman 2014b, pp. 99–100). Tolerating a strange element also takes place in relation to others (Dalibert 2015; Hudak et al. 2007; Slatman 2014b). For example, how the recipient of a mechanical heart valve deals with the clicking sounds of the valve depends on both how they and others relate to these sounds. For most people these sounds move to the background of the awareness, i.e. become transparent, while others may become anxious or are instead reassured by hearing these sounds. For example, a partner who hears the clicking sounds might be unable to sleep because they want to make sure the person with the heart valve is fine. The responses of others can influence how the recipient relates to their implant and tolerates the implant and could thus affect the embodiment process.

Third, by mirroring ourselves to others, we aim to conform to certain societal norms, and this also affects our ability to tolerate the strange (Slatman 2014b). For example, Lucie Dalibert describes a case study of a woman for whom the visibility of a pulse generator that was implanted just under the skin negatively affects her embodiment process (Dalibert 2015). The woman sees her body through the eyes of her husband and concludes that her body is not attractive anymore although her husband thinks otherwise (he says he does not mind the implant). The visibility of the implant does not fit within the dominant societal norm of women having soft and smooth skin (Dalibert 2015) and hinders the woman’s ability to affectively tolerate the presence of the implant under her skin. Thus, how the body appears to other people matters for how it appears to oneself (Slatman 2014a). Bodies with implants are embedded in networks of relationships and this embeddedness is important for the liveability of the implanted technology (Dalibert 2015).

Fourth, in the context of organ and limb transplantations, the ‘other’ in the role of organ donor is particularly important for tolerating the strange. In organ and limb transplants the matter which is added to the body is reanimated or appropriated for a second time (Slatman 2014b). The recipient of a donor organ or body part must deal with the notion that the lifeless matter from another person has become part of their still-living body. This can evoke feelings of alienation and hinder the process of affective tolerance. Phenomenological empirical studies on heart and liver transplants indicate that recipients acknowledge the role of the donor in their process of tolerating the strange (Forsberg et al. 2000; Mauthner et al. 2015; Sadala & Stolf 2008). For example, recipients of heart transplants express a sense of interconnectedness with the donor (Mauthner et al.), believing that the donor’s essence lives on within them. This experience can lead to distress and challenges to one’s sense of identity (Mauthner et al. 2015). In some research, transplant recipients even report a sense of adopting certain characteristics of their donors (Bunzel et al. 1992; Inspector et al. 2004; Mauthner et al. 2015). Affective tolerance in case of organ and limb transplantation therefore requires that the recipient relates to the role of the donor as a unique ‘other’.

Notably, while we separate functional adjustments and affective tolerance as conditions for tolerating the strange for heuristic purposes, the two conditions are in fact interconnected, just as the body schema and body image are interconnected. During interactions with others, our body schema instinctively situates the body (Zeiler 2009). In this engagement with others and the world, new bodily skills can be required, and the body schema sometimes needs to be revised (Zeiler 2009). For instance, the woman with the pulse generator has the feeling that her body is no longer attractive to her husband (affective tolerance), affecting how she moves and carries herself in his presence. This shift in body image requires new skills and adjustments to her body schema (functional adjustments). In short, as conditions for tolerating strange elements in the body, functional adjustment and affective tolerance are interconnected.

## Embodiment of regenerative implants

In this section, we shortly compare regenerative implants to regular implants and organ and limb transplants, and then apply the insights from the existing phenomenological and empirical literature on embodiment and regular implants and organ and limb transplants to better understand the experience of living with regenerative implants.

### Regenerative implants vs. regular implants and organ and limb transplants

Regenerative implants differ from regular implants and organ and limb transplants because they are internal (rather than external and detachable like prostheses), synthetic (rather than biologically derived, like hand transplants) and, importantly, biodegradable and regenerative (rather than inert, like hip implants).

Like regular implants, regenerative implants are of strange (foreign) origin, and exert influence over the body without our control. Yet in contrast to regular (inert) implants, regenerative implants are transformed from strange matter into living tissue. Moreover, in contrast to regular (inert) implants, regenerative implants continuously act on and within the body (Parry 2017): the implant materials sense and respond to the surrounding tissues, and dynamically interact and ‘communicate’ with the living tissues (De Kanter et al. 2023b). These biodegradative and regenerative processes are ongoing interactions between the implant and body that cannot be actively controlled by the recipient, and which could reinforce the strangeness of the body.

Organ and limb transplants already involve living tissue (from a donor). In this aspect, regenerative implants and organ and limb transplants share a commonality, as they both eventually become interwoven in the body as living material, and actively act on and intervene with the body. Yet, they also differ. Recipients of donor organs have to navigate the complex relationship with the donor, which is absent in the case of regenerative implants. Moreover, when transplanted, the donor organ, functioning as the object, and the recipient’s body, serving as the subject, remain distinguishable by the body as foreign material. This is evident in the body’s ongoing rejection of the donor organ (whether or not suppressed by the lifelong intake of immunosuppressive drugs). In contrast, regenerative implants transform into autologous body tissue, avoiding the risk of immune rejection.

In our discussion of tolerating regenerative implants below, we will focus mainly on the biodegradable and regenerative properties that distinguish them from regular implants and organ and limb transplants.

### Tolerating regenerative implants

Regenerative implants are designed to integrate intimately into the living tissues of the human body. The implant is biodegradable, so that it dissolves in the body, and it is regenerative, so that cells grow into the material and eventually transform into living tissue. This means that, assuming that regenerative implants will be successful in a strictly medical sense^7^ (i.e. the tissue will successfully regenerate), the implant and the surrounding tissues of the body become one. The implant as an object fuses with the body as Körper. The strangeness of the implant joins the strangeness of the body, and over time the two become indistinguishable. We hypothesise that this process could affect the embodied experience of living with a regenerative implant in two distinct ways.

On the one hand, as we saw in the previous section, the experience of our body as Körper is always part of the Leib-experience, of the me-ness of the body. Therefore, the fusion of the regenerative implant with the Körper could help to experience the implant as part of the Leib. By being transformed into living tissue, the implants become part of the fleshiness of the body. This might allow for the implants to be lived as a nearly (though never completely) inseparable part of the lived body (Leib), which could help us to perceive the implant as our own. In this case, the implant does not remain merely an object, but becomes a subject also, just as the Leib (De Kanter et al. 2023a). The implant becomes ‘me’ and might therefore be more easily tolerated. The experience of the implant as me would allow for lived unity between having and being a body, and this could foster the embodied experience of living with an implant.

On the other hand, the properties of regenerative implants could also have a different, opposite, effect on the embodied experience of living with an implant. The fusion of the implant—a foreign technology—with the Körper could emphasize the object-like strangeness of the physical body. The dynamic interactions of the regenerative implant with the physical body, which cannot be actively controlled by the recipient, could reinforce the strangeness of the body. This could detract me-ness from the Leib experience and challenge the lived unity between having and being a body, between Körper and Leib. Eventually, this could lead to estrangement of both the implant and the body: the body fused with the implant could become a stranger to the person itself. As a result, the implant might not be tolerated by the recipient, which could hinder the therapeutic process (De Kanter et al. 2023b). Also, if the tissue is successfully regenerated, the recipient may still experience a sense of unease or discomfort with their body, and the aims of the treatment, e.g. to improve one’s independence and freedom of movement, might not be fulfilled.

With Slatman, we may expect that whether regenerative implants will allow for me-ness, or cause bodily estrangement depends on the functional or affective conditions to tolerating the strange. In what follows, we briefly discuss how both conditions could affect the embodiment of regenerative implants.

#### A. Functional adjustment of regenerative implants

With regards to functional adjustment, regenerative implants hold the potential to restore or improve sensations including proprioception, kinaesthetic sensations, and a sense of touch. When the implant becomes replaced by living tissue, it also becomes innervated and thus perceivable through the nervous system from within, i.e. no longer only through the surrounding tissues. Such localized sensations would allow for incorporating the implant into the body schema, so that it can be a lived part of the body as we use it to navigate the world in everyday life. For a regenerative jawbone implant, this would mean that one could chew food without having to think about it consciously. For a regenerative heart valve implant, even if lived unconsciously, kinaesthetic sensations could also improve functionality. For example, like native valve tissue, regenerated valve tissue might be able to tighten in response to high blood pressure if kinaesthetic sensations are restored.

#### B. Affective tolerance of regenerative implants

With regard to affective tolerance, regenerative implants should allow the recipient to identify with their body image. This is a process that takes place in relation to oneself (self-perception), to close others (interpersonal relationships) and to social norms. Regenerative implants can change someone’s body from the outside, even if they are brought within the body. By altering the external appearance of the body, regenerative implants could (positively or negatively) affect self-perception, interpersonal relationships and conformity to social norms, which influences the extent to which individuals identify with their body image. Regarding self-perception, recipients of a regenerative jawbone implant, that could alter one’s visual appearance, might require time to get used to their new mirror image. In relation to others, if a regenerative valve implant draws less attention to itself in terms of sound and visibility compared to regular implants (such as mechanical valves that make a clicking sound), this might make it easier to relate to others. And in relation to social norms, such as beauty standards, changes to one’s facial appearance due to a jawbone implant could influence how one is perceived by others, e.g. one might be judged as being dumb or scary if one’s face ends up asymmetrical.

Taken together, we expect that regenerative implants—because they are transformed into body tissue—could be experienced as part of the body entirely, or could lead to estrangement from the own body. We showed that, based on the available literature, we may expect that functional adjustments and affective tolerance are important conditions for tolerating regenerative implants as part of the body. However, these conditions are similarly applicable to regular implants as to regenerative implants and do not get to the core of describing how fusion of regenerative implants with the Körper might affect the Leib experience. Nonetheless, our analysis above suggests that regenerative implants might constitute an experience that is qualitatively different from regular implants and organ transplants, as we will explore in more detail below.

## Embodiment and ‘entanglement’ in the context of regenerative implants

In this section, we will demonstrate that current concepts and conditions in the phenomenological literature fall short in describing the interwovenness and intimate relationship of regenerative implants and the lived body. We will argue that regenerative implants call for a new concept and propose ‘entanglement’ as a phenomenological concept to conceptualise the embodied experience of living with regenerative implants more accurately.

### Interwovenness and the intimate relationship between regenerative implants and the body

Based on section “Embodiment of regenerative implants”, there is reason to expect that regenerative implants might constitute an experience that is qualitatively different from regular implants and organ transplants, and that is related to the fusion of the implant with the body (Körper). This fusion has two relevant implications: regenerative implants become *interwoven* with the lived body, and this relationship is particularly *intimate*. The first implication is related to the material properties of regenerative implants and the second implication is about the inter-action of the implant with the body.

First, regular implants are inert and their presence in the body is durable. Therefore, the material boundary between body and technology remains clear. Likewise, in organ and limb transplants the recipient’s body marks the donor organ as foreign tissue, and the material boundary between the recipient’s body and the donor organ remains defined. Regenerative implants by contrast bring a new dimension of interwovenness between humans and technology in which the human body merges with the technology. The subject and object cannot be distinguished anymore and become interdependent because of the regenerative and biodegradable capacities of the implant. The interwovenness between humans and technology might foster the embodied experience of the recipient (i.e. reduce alienation compared to regular implants or transplants) or can lead to alienation of the body.

Second and relatedly, the relationship between regenerative implants and the body is particularly intimate, because the implants *act* and *intervene* in the body and cannot be understood outside of this relationship with the body (Dalibert 2015). While regular implants do not transform into body tissue and organ and limb transplants have to navigate a unique relationship with the donor, regenerative implants act and intervene on a different level. This intimate relationship between regenerative implants and the body can lead to a sense of diminished control for the recipient or foster the incorporation process as it becomes body tissue.

The phenomenological literature that we discussed in Sect. "Embodiment and the experience of living with regular implants" lacks the concepts to capture this new dimension of interwovenness and the intimate relationship between regenerative implants and the body. Slatman’s work on tolerating strange elements as part of the body points into the right direction, as we showed in Sect. "Embodiment of regenerative implants". Yet her proposal for functional adjustment and affective tolerance as conditions for tolerating the strange are similarly applicable to regenerative implants as they are to regular implants, and do not get the essence of describing the new intimate relationship between humans and technology in which humans and technology fuse. The way humans and regenerative implants merge transcends the mere functional and affective dimensions because of the biodegradable and regenerative capacities of the implants.

This lack of concepts is not surprising because regenerative implants are a novel technology, and existing phenomenological literature is based on regular implants and organ and limb transplants. Although resorbable stiches have been available for a while, synthetic implants that stimulate the body to regenerate larger tissues or even organs are only emerging now. This development asks for a new concept that can capture the embodied experience of living with regenerative implants. Such a concept is needed to grasp, think through, and analyse what is particular about this experience. We propose that this new dimension of interwovenness and the intimate relationship between humans and technology should be understood as *entanglement*.

### Entanglement as a phenomenological concept

‘Entanglement’ as a concept has been used in many fields such as physics and the social sciences and humanities. Over the past decades, the social sciences and humanities saw a notable shift in which renewed emphasis on the importance of objects was placed (Hodder 2014). From actor-network theory to anthropological accounts of materiality, there is agreement that subject and object, mind and matter, humans and things co-constitute each other (Hodder 2014). All these accounts have emphasized the entangled nature of human existence and social life with material objects^8^ (Hodder 2014).

The concept of ‘entanglement’ is used to describe the relational nature of humans and things. According to Hodder, entanglement can be defined as “the dialectic of dependence and dependency between humans and things” (Hodder 2014, p. 20). In short, entanglement refers to the inescapable and intimate relationship between humans and non-human materialities (including technologies) which co-constitute each other in a dynamic manner (Dale and Latham 2014; Hodder 2014).

We propose that entanglement can be used in a similar manner for the phenomenological analysis of the relationship of humans with technologies, and particularly for understanding the interwovenness and intimate relationship of recipients with their regenerative implants. The knowledge that the body is entangled with an implanted technology and that body and technology merge can lead to alienation from the body or, conversely, could promote the embodied experience as the implant eventually becomes the body’s own tissue.

We hypothesize that accepting entanglement is a third condition – in addition to functional adjustment and affective tolerance – for tolerating the strange when it comes to implantable technologies that fuse with the body. In this context, entanglement serves as a multifaceted phenomenon. On the one hand, entanglement can manifest as a barrier to tolerating the regenerative implant, because of the possible associated experience of alienation. To overcome this barrier, the recipient needs to accept the entanglement process to tolerate a regenerative implant as part of the body. This acceptance requires an active and conscious effort. On the other hand, entanglement can serve as a facilitator in tolerating the strange as it becomes the body’s own tissue. Here, the recipient need not take active or conscious steps, as the integration unfolds naturally.

### Entanglement in the context of post-phenomenology

Now that we have explored entanglement as a phenomenological concept, we contextualize this concept within the framework of post-phenomenology. Post-phenomenology comprehensively analyses the fusion of humans and technological artifacts, including implantable technologies, and therefore has a number of commonalities with our analysis that are worth exploring.

Post-phenomenology can be defined as a field that investigates technology by focusing on the relation between human beings and technological artifacts, with an emphasis on how technologies shape the relations of humans with the world (Rosenberger and Verbeek 2015). It combines philosophical analysis with empirical investigation. Ihde laid the foundations for the exploration of various human-technology relations, including the already briefly mentioned embodiment relation. More recently, post-phenomenologists have argued that to fully grasp the profound experiences and effects of implantable devices, the concepts of embodiment, mediation, transparency and trade-offs between these concepts need to be expanded (Rosenberger and Verbeek 2015). In line with this argument, Peter-Paul Verbeek has introduced the concept of ‘cyborg relations’, as an extension of the embodiment relation, to redefine the relation between humans and implantable technologies (Verbeek 2008). In cyborg relations, humans and technology become one and relate to the world together (hybrid intentionality) (Verbeek 2008).

We agree that there is a need for new concepts to understand the intimate relationship between humans and implantable technologies. Like Verbeek’s notion of cyborg relations, entanglement signifies a deep intertwining of humans and implantable technologies, going beyond the idea of mere coexistence. In entanglement, humans and implantable technologies become intricately interwoven and their relationship becomes particularly intimate, with their boundaries and identities blending into a new cohesive whole. This understanding of entanglement allows us to explore the complexities of human-technology relations, acknowledging the inseparability of human and implantable technologies.

At the same time, our concept of entanglement differs from post-phenomenology and particularly Verbeek’s understanding of cyborg relations. This difference derives from the criticism of Verbeek’s theory for potentially overlooking the significance of the lived experience, and the experience of living in a particular body (Dalibert 2014). Therefore, our proposal to understand this new dimension of interwovenness and the intimate relationship between humans and technology in terms of ‘entanglement’ differs because we do not aim to capture the phenomenon in an abstract sense. Instead, our approach focuses on how people might experience living with regenerative implants, with particular attention to their relationship with their body, rather than focusing on their relation with the world in its entirety.

## Discussion

The primary objective of our analysis was to explore how regenerative implants may affect the embodied experience of recipients and explore and improve the phenomenological concepts necessary to comprehend this process.

In the first part of this paper, we drew lessons from the current phenomenological literature regarding the experience of living with regular implants and organ and limb transplants. In the second part of this paper, we applied these lessons to explore how future implant recipients might experience living with regenerative implants. We showed there is reason to expect that regenerative implants will constitute an experience that is qualitatively different from regular implants and organ and limb transplants, specifically due to the fusion of the implant with the Körper, which is also likely to affect the Leib experience. In the third part of this paper, we argued that the existing phenomenological literature lacks the necessary conceptual framework to comprehend this unique experience. Hence, we introduced the concept of entanglement as a third condition for tolerating the strange. As a phenomenological concept, entanglement describes the intimate interconnectedness of people living with technologies that fuse with the body. Entanglement can either pose a barrier for recipients to accept the implant as part of their body, or it can make to toleration process easier as the implant becomes the body’s own tissue.

Notably, in our analysis we departed from the traditional phenomenological approach by exploring the future implications of a technology still under development. Phenomenology is traditionally concerned with experiences in the present and the past, because of its focus on the lived experience. We instead applied phenomenological concepts to explore how regenerative implants might affect the lived experiences of people who will be living with such implants in the future. We think such a forward-looking approach is justifiable because regenerative implants are still under development and therefore empirical research into the lived experiences of people with such implants is not yet possible. Moreover, much can be learned from existing phenomenological analyses of the lived experience of health and illness, as well as the experience of living with implantable technologies. Finally, there is reason to collect these lessons now as they can help to shape the design of the regenerative implants as well as the pre- and post-surgical care of recipients of those implants. Our endeavour in this paper might therefore also be read as an exploration of how to use insights from traditional and empirical phenomenology for a forward-looking exploration of lived experiences with new and emerging technologies.

## Considerations for future research

Once regenerative implants become available in clinical practice, our theoretical analysis and proposal for ‘entanglement’ can guide empirical research to examine the actual lived experience of implant recipients. As such, these insights can help to make sure that regenerative implants that cure the physical body will also improve the lived and everyday experience of individuals who rely on these implants. This could also facilitate patient-centred treatment and care. Additionally, qualitative empirical research (e.g. interview studies based on interpretative phenomenological analysis) into the lived experience of people living with regenerative implants is important to test the practical value and empirical validity of our proposal for entanglement, and to deepen understanding of the concept.

In our analysis, we used two examples, namely regenerative jawbone implants and regenerative heart valves, to illustrate how these applications can have distinct impacts on the embodied experience. It is crucial to conduct future research to explore the diverse ways in which the embodied experience can be influenced across various applications of regenerative implants. For instance, we recognize that interventions in the heart hold a different cultural, emotional, and possibly spiritual significance than alterations to the head or joints, and that this significance might differ across cultures and religions. Understanding these nuances is necessary for understanding what it will be like to live with a regenerative implant.

Moreover, we acknowledge that lived experiences vary between individuals, as each implant recipient possesses unique perceptions of the world, themselves, and their implant. Consequently, understanding embodiment requires attending to the unique circumstances and experiences of each individual. While some recipients may find it easier to adapt to the new situation of living with a regenerative implant, others may encounter more challenges and difficulties. Nonetheless, it is evident that attention should be paid to the lived experience of recipients both in the design process of the regenerative implants, as during pre- and post-surgical care of the recipient, because embodiment is likely to significantly impact the therapeutic outcomes associated with regenerative implants. This is especially true for cases where somatic symptoms are prevalent for which there is no medical explanation available. As such, attending to the individual’s lived experience can improve the overall therapeutic success of regenerative implants.

## Footnotes

1 In the bioengineering literature, this approach is referred to as in situ tissue engineering. The manufacturing of these implants often relies on 3D printing and the implants may be personalized to fit the unique body of each recipient.

2 An exception is Mechteld Derksen’s phenomenological analysis of tissue engineered heart valves (Derksen and Horstman 2008; Derksen 2008). Derksen’s analysis is valuable in criticizing the assumptions underlying the narrative that engineers have constructed regarding these heart valves (which presents the heart valves as natural and transparent). Yet Derksen’s analysis lacks a positive hypothesis of how tissue engineered heart valves will be lived by people receiving the valves.

3 We use the term ‘regular implants’ to refer to implants used in regular clinical practice, such a knee and hip implants, that are made from synthetic materials (such as metal, ceramics and certain polymers) that are inert (i.e. that do not interact with the body) and non-degradable.

4 In some conceptualizations, embodiment can refer to merely the fact that people are always embodied beings, embodied minds. Instead, following Ihde, we use embodiment to refer to how the body is experienced. For example, he writes “As a reader of much feminist literature (…) I find a set of issues that revolve around how one experiences embodiment” (Ihde 2002).

5 Slatman herself does not provide an explicit definition or explanation of the functional and affective conditions to tolerating strangeness, nor does she explicate clearly how both conditions relate to other parts of the theoretical exposition in the rest of her book. Instead, she mainly illustrates the conditions through the discussion of a number of case studies that function as examples (Slatman 2014b).

6 Our interpretation of Slatman differs at this point from the interpretation of Tbavaldany et al. who have interpreted functionality and proprioceptive feedback as two separate conditions for tolerating the strange (Tbalvaldany et al. 2018).

7 We do not discuss the situation in which a regenerative implant turns out to be unsuccessful, e.g., if the implant would not sufficiently break down, not lead to successful regeneration, and/or lead to unwanted tissue-growth (tumor tissue). Such situations would significantly harm the recipients and would therefore most certainly cause significant disruptions in the lived unity of the body-mind-world system, as does any experience of severe illness.

8 In his final, uncompleted, work, The Visible and The Invisible, Merleau-Ponty (1968) moves even further and abandons the subject-object distinction by introducing intertwinement/chiasm. He restructures an ontology in which everything is flesh (la chair), and everything unfolds in the flesh, both subject and object, both the lived body as the world (Merleau-Ponty 1968). Given that our phenomenological analysis involves a specific case of intertwinement, where technology and the body fuse, and we do not discuss all subject-object relations in the world as Merleau-Ponty does, we do not employ his terminology but instead adopted the term entanglement.
